# Crystal structure and Hirshfeld surface analysis of 2-[(2-oxo-2*H*-chromen-4-yl)­oxy]acetic acid dimethyl sulfoxide monosolvate

**DOI:** 10.1107/S2056989019009447

**Published:** 2019-07-09

**Authors:** S. Syed Abuthahir, M. NizamMohideen, V. Viswanathan, M. Govindhan, K. Subramanian

**Affiliations:** a PG & Research Department of Physics, The New College (Autonomous), University of Madras, Chennai 600 014, Tamil Nadu, India; b Department of Biophysics, All India Institute of Medical Science, New Delhi 110 029, India; c Department of Chemistry, Anna University, Chennai 600 025, India; d Orchid Chemicals & Pharmaceuticals Ltd, R&D Centre, Sholinganallur, Chennai 600 119, India

**Keywords:** crystal structure, chromen, acetamide, pyran, dimethyl sulfoxide, hydrogen bonding, C—H⋯π inter­actions, offset π–π inter­actions, Hirshfeld surface analysis

## Abstract

The title compound, C_11_H_8_O_5_·(CH_3_)_2_SO, is a new coumarin derivative. The asymmetric unit contains two coumarin mol­ecules (*A* and *B*) and two di­methyl­sulfoxide solvent mol­ecules (*A* and *B*). The dihedral angle between the pyran and benzene rings in the chromene moiety is 3.56 (2)° for mol­ecule *A* and 1.83 (2)° for mol­ecule *B*. In mol­ecule *A*, the dimethyl sulfoxide sulfur atom is disordered over two positions with a refined occupancy ratio of 0.782 (5):0.218 (5). In the crystal, mol­ecules are linked by O—H⋯O hydrogen bonds, forming chains running along the *c-*axis direction. The chains are linked by C—H⋯O hydrogen bonds, forming layers parallel to the *ac* plane. In addition, there are also C—H⋯π and π–π inter­actions present within the layers. The inter­molecular contacts in the crystal have been analysed using Hirshfeld surface analysis and two-dimensional fingerprint plots, which indicate that the most important contributions to the packing are from H⋯H (33.9%) and O⋯H/H⋯O (41.2%) contacts.

## Abstract 2

The crystal structure of 2-[(2-oxo-2*H*-chromen-4-yl)­oxy]acetic acid di­methyl­sulfoxide monosolvate is described and the inter­molecular contacts in the crystal analysed using Hirshfeld surface analysis and two-dimensional fingerprint plots.

## Chemical context   

Coumarin and its derivatives represent one of the most active classes of compounds, possessing a wide spectrum of biological activity. The synthesis, and pharmacological and other properties of coumarin derivatives have been studied intensively and reviewed (Syed Abuthahir *et al.*, 2019 ▸; Kumar *et al.*, 2015 ▸; Kubrak *et al.*, 2017 ▸; Srikrishna *et al.*, 2018 ▸; Venugopala *et al.*, 2013 ▸). Many of these compounds have proven to be active as anti­bacterial, anti­fungal, anti-inflammatory, anti­coagulant, anti-HIV and anti­tumor agents (Govindhan, Subramanian, Chennakesava Rao *et al.*, 2015 ▸; Govindhan, Subramanian, Sridharan *et al.*, 2015 ▸). Sulfur-containing isocoumarins (Henderson & Hill, 1982 ▸), fluorine-containing isocoumarins (Babar *et al.*, 2008 ▸) and chlorine-containing isocoumarins (Abid *et al.*, 2008 ▸) have also been studied. In view of the importance of their natural occurrence, biological, pharmacological and medicinal activities, and their use as synthetic inter­mediates, we have synthesized the title derivative 2-[(2-oxo-2*H*-chromen-4-yl)­oxy]acetic acid dimethyl sulfoxide monosolvate, and report herein on its crystal structure and Hirshfeld surface analysis.

## Structural commentary   

**Figure 1:**
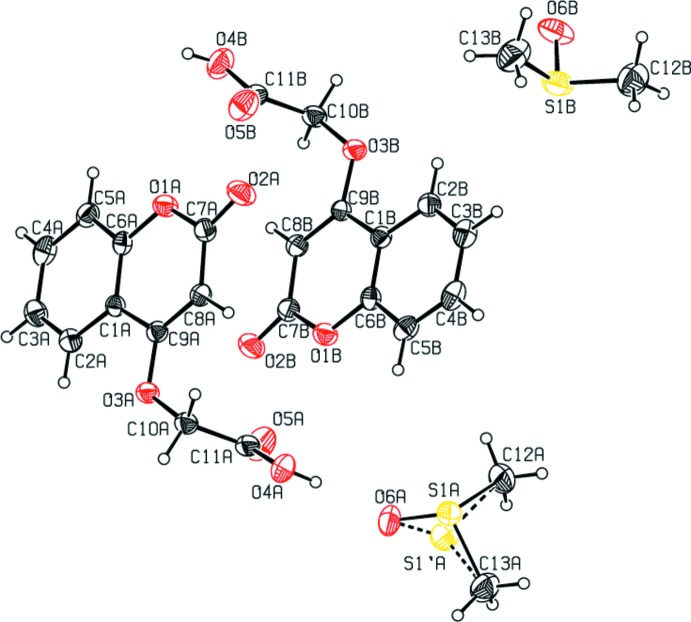
The mol­ecular structure of the compound, with the atom labelling. Displacement ellipsoids are drawn at the 30% probability level.

**Figure 2:**
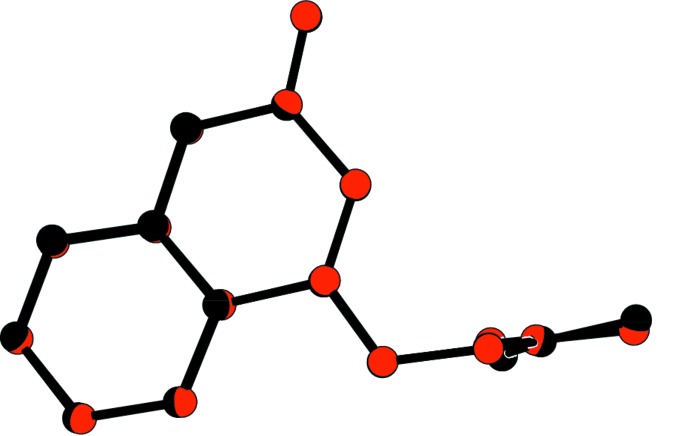
A view of the mol­ecule overlay of inverted mol­ecule *B* (red) on mol­ecule *A* (blue), with an r.m.s. deviation of 0.126 Å.

The mol­ecular structure and conformation of the two independent mol­ecules, *A* and *B* in the asymmetric unit, are shown in Fig. 1 ▸. The bond lengths and angles in both mol­ecules are very similar. The normal probability plot analyses (Inter­national Tables for X-ray Crystallography, 1974, Vol. IV, pp. 293–309) for both bond lengths and angles show that the differences between the two symmetry-independent mol­ecules are of a statistical nature. The structural overlay of the two mol­ecules is shown in Fig. 2 ▸ (r.m.s. deviation = 0.098 Å).
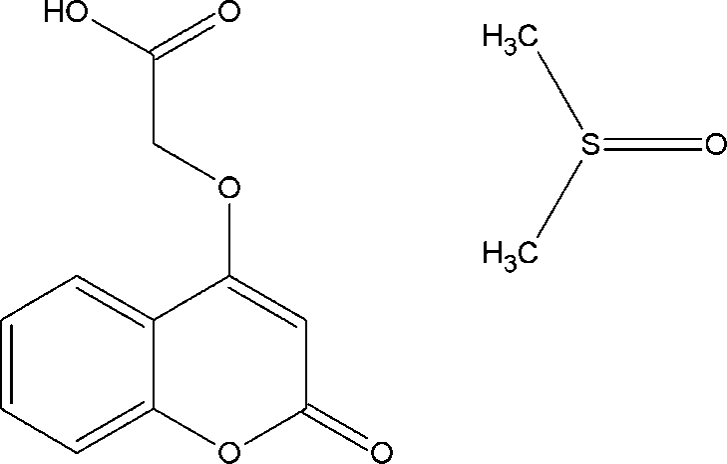


The 1*H*-isochromene moiety is planar (r.m.s. deviation = 0.001 Å for mol­ecule *A* and 0.015 Å for mol­ecule *B*) and atoms O2*A* and O2*B* deviate from this mean plane by 0.007 (3) and 0.039 (3) Å, respectively. The dihedral angle between the pyran and benzene rings in the chromene moiety is 3.56 (16)° for mol­ecule *A* and 1.83 (16)° for mol­ecule *B*; this value is in agreement with those found in analogous coumarin derivatives (Dobson & Gerkin, 1996 ▸; Kokila *et al.*, 1996 ▸). In mol­ecule *A*, the dimethyl sulfoxide sulfur atom is disordered over two positions with refined occupancies of 0.782 (5) and 0.218 (5).

The title compound exhibits structural similarities with those of two new coumarin derivatives: 2-(4-{2-[(2-oxo-2*H*-chromen-4-yl)­oxy]acet­yl}piperazin-1-yl)acetamide (Govin­d­han, Subramanian, Chennakesava Rao *et al.*, 2015 ▸) and *N*-(2,4-di­meth­oxy­benz­yl)-2-[(2-oxo-2*H*-chromen-4-yl)­oxy]acetamide (Syed Abuthahir *et al.*, 2019 ▸).

## Supra­molecular features   

**Figure 3:**
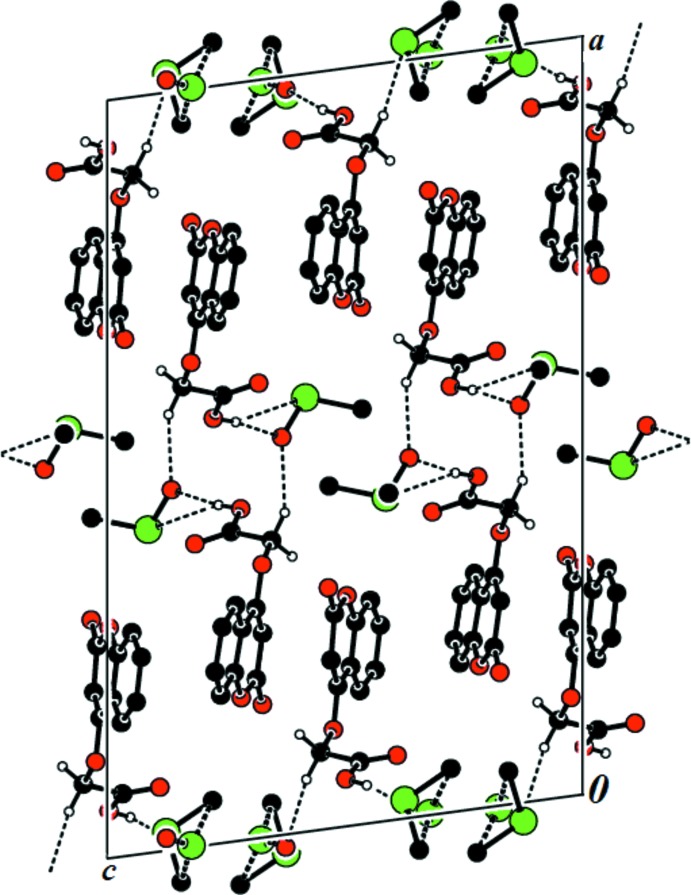
A view along the *b* axis of the crystal packing of the title compound. The hydrogen bonds (Table 1 ▸) are shown as dashed lines, and H atoms not involved in hydrogen bonding have been omitted.

The crystal structure features O—H⋯O and C—H⋯O hydrogen bonds (Table 1 ▸; Fig. 3 ▸). In the crystal, the *A* and *B* mol­ecules are linked by O—H⋯O hydrogen bonds, forming chains running along the *c-*axis direction. The chains are linked by C—H⋯O hydrogen bonds, forming layers parallel to the *ac* plane. C—H⋯π (Table 1 ▸) and π–π inter­actions are present within the layers. The observed π–π inter­actions involve the pyran ring of the chromene ring system and the benzene ring [*Cg*1⋯*Cg*3^iv^ = 3.864 (2), *Cg*1⋯*Cg*4^iv^ = 3.509 (2) and *Cg*2⋯*Cg*3^iv^ 3.572 (2) Å where *Cg*1, *Cg*2, *Cg*3 and *Cg*4 are the centroids of rings O1*A*/C1*A*/C6*A*–C9*A*, C1*A*–C6*A*, O1*B*/C1*B*/C6*B*–C9*B*, and C1*B*–C6*B*, respectively; symmetry code: (iv) *x*, 1 + *y*, *z*].

## Hirshfeld surface analysis   

A recent article by Tiekink and collaborators (Tan *et al.*, 2019 ▸) reviews and describes the uses and utility of Hirshfeld surface analysis (Spackman & Jayatilaka, 2009 ▸), and the associated two-dimensional fingerprint plots (McKinnon *et al.*, 2007 ▸), to analyse inter­molecular contacts in crystals. The various calculations for the title compound were performed with *CrystalExplorer17* (Turner *et al.*, 2017 ▸).

**Figure 4:**
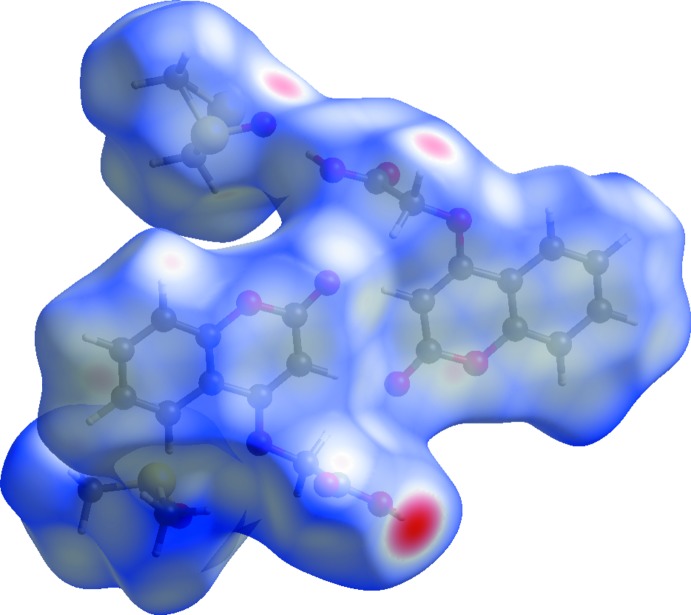
The Hirshfeld surface of the title compound, mapped over *d*_norm_.

**Figure 5:**
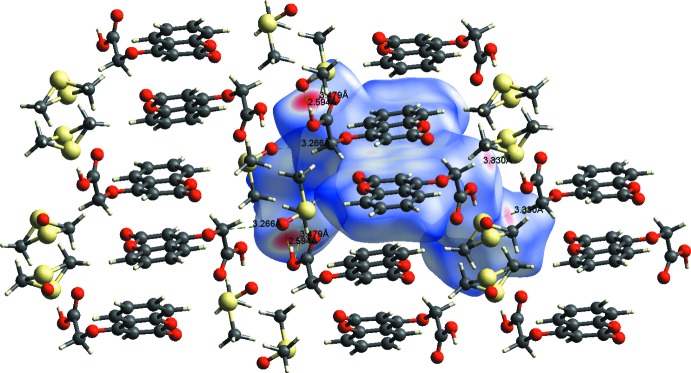
A view of the Hirshfeld surface mapped over *d*_norm_, showing the various inter­molecular contacts in the crystal of the title compound.

The Hirshfeld surface of the title compound mapped over *d*_norm_ is shown in Fig. 4 ▸, and the inter­molecular contacts are illustrated in Fig. 5 ▸. They are colour-mapped with the normalized contact distance, *d*_norm_, ranging from red (distances shorter than the sum of the van der Waals radii) through white to blue (distances longer than the sum of the van der Waals radii). The *d*_norm_ surface was mapped over a fixed colour scale of 0.774 (red) to 1.381 (blue) for the title compound, where the red spots indicate the inter­molecular contacts involved in the hydrogen bonding.

**Figure 6:**
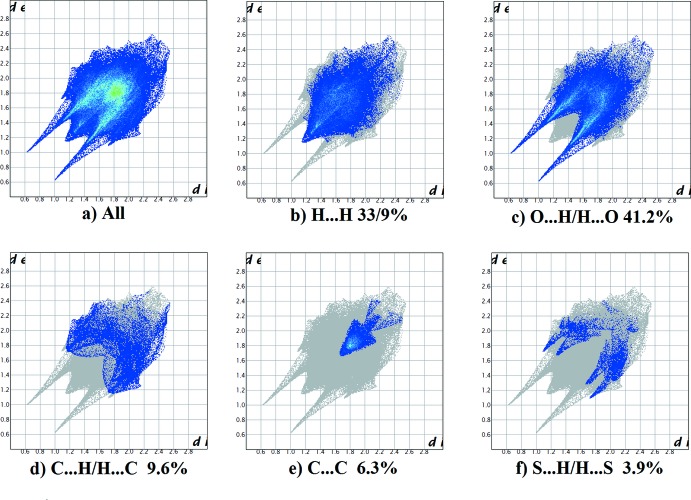
(*a*) The full two-dimensional fingerprint plot for the title compound, and those delineated into (*b*) H⋯H, (*c*) O⋯H/H⋯O, (*d*) C⋯H/H⋯C, (*e*) C⋯C and (*f*) S⋯H/H⋯S contacts.

The fingerprint plots are given in Fig. 6 ▸. They reveal that the principal inter­molecular contacts are H⋯H at 33.9% (Fig. 6 ▸ *b*) and O⋯H/H⋯O at 41.2% (Fig. 6 ▸ *c*), followed by the C⋯H/H⋯C contacts at 9.6% (Fig. 6 ▸ *d)*, C⋯C contacts at 6.3% (Fig. 6 ▸ *e*) and S⋯H/H⋯S contacts at 3.9% (Fig. 6 ▸ *f*).

## Database survey   

A search of the Cambridge Structural Database (Web CSD version 5.39; March 9, 2018; Groom *et al.*, 2016 ▸) gave more than 35 hits for both linear and angular pyran­ocoumarin (psoralene class) structures. They include seselin (amyrolin) [refcodes AMYROL (Kato, 1970 ▸) and AMYROL01 (Bauri *et al.*, 2006 ▸)], 2,3-dihy­droxy-9-hy­droxy-2(1-hy­droxy-1-methyl­eth­yl)-7*H*-furo-[3,2-*g*][1]-benzo­pyran-7-one monohydrate (FUGVOS; Thailambal & Pattabhi, 1987 ▸), bromo­hydroxy­seselin (XARQAL; Bauri *et al.*, 2017*a* ▸), di­bromo­mometh­oxy­seselin (VAPKOP; Bauri *et al.*, 2017*b* ▸), and a number of structures with various substituents at C3 and C4, many of which are natural products.

Intra­molecular C—H⋯O short contacts similar to those in the title compound are found in five compounds in the CSD: 1-(1-pyrrolidinylcarbon­yl)cyclo­propyl sulfamate (LISLAB; Morin *et al.*, 2007 ▸), 2-[3′-(4"-chloro­phen­yl)-4′,6′-di­meth­oxy­indol-7′-yl]glyoxyl-1-pyrrolidine (PEQHAU; Black *et al.*, 1997 ▸), [2-hy­droxy-5-(2-hy­droxy­benzo­yl)phen­yl](pyrrolidin-1-yl)methanone (QIBBEJ; Holtz *et al.*, 2007 ▸), 2-meth­oxy-1-(1-pyrrolidinylcarbon­yl)naphthalene (SIHNAZ; Sakamoto *et al.*, 2007 ▸) and (4*S*,5*S*)-4,5-bis­(pyrrolidinylcarbon­yl)-2,2-dimethyl-1,3-dioxolane (TAJDIR; Garcia *et al.*, 1991 ▸).

## Synthesis and crystallization   

A solution of lithium hydroxide (0.24 g, 1.2 mol eq.) in water (4 mL) was added to ethyl 2-(2-oxo-2*H*-chromen-4-yl­oxy) acetate (2.0 g, 1.0 mol eq.) in THF (10 mL) at 273 K and stirred at 273 K for 1 h. Completion of the reaction was confirmed by TLC (mobile phase ethyl acetate/hexa­ne) and THF was distilled off using a rotavapor. The obtained solution was washed with ethyl acetate (20 mL). The aqueous layer was acidified with 2*N* HCl (pH 1.0–2.0) and the obtained solid was filtered, washed with hexane and dried under vacuum to give as white solid. The purified compound was recrystallized using dimethyl sulfoxide as solvent.

## Refinement   

Crystal data, data collection and structure refinement details are summarized in Table 2 ▸. The H atoms were positioned geometrically and constrained to ride on their parent atoms: C—H = 0.93–0.97Å with *U*_iso_(H) = 1.5*U*_eq_(C-meth­yl) or 1.2*U*_eq_(C) for other H atoms. In mol­ecule *A*, the sulfur atom of the sulfinyldi­methane group is disordered over two positions with refined occupancies of 0.782 (5) and 0.218 (5). In the final cycles of refinement, five outliers were omitted.

## Supplementary Material

Crystal structure: contains datablock(s) global, I. DOI: 10.1107/S2056989019009447/vm2218sup1.cif

Structure factors: contains datablock(s) I. DOI: 10.1107/S2056989019009447/vm2218Isup2.hkl

Supporting information file. DOI: 10.1107/S2056989019009447/vm2218Isup3.cml

CCDC reference: 1891495

Additional supporting information:  crystallographic information; 3D view; checkCIF report

## Figures and Tables

**Table 1:** Hydrogen-bond geometry (Å, °) *Cg*2 is the centroid of the C1*A*–C6*A* ring.

*D*—H⋯*A*	*D*—H	H⋯*A*	*D*⋯*A*	*D*—H⋯*A*
O4*A*—H4*A*1⋯O6*A*	0.82	1.82	2.621 (5)	167
O4*B*—H4*B*1⋯S1*B* ^i^	0.82	2.70	3.479 (3)	159
O4*B*—H4*B*1⋯O6*B* ^i^	0.82	1.78	2.595 (4)	169
C10*B*—H10*A*⋯O2*A*	0.97	2.49	3.423 (4)	161
C10*B*—H10*B*⋯O6*B* ^ii^	0.97	2.37	3.266 (4)	153
C10*A*—H10*C*⋯O6*A* ^iii^	0.97	2.38	3.330 (5)	165
C10*A*—H10*D*⋯O2*B*	0.97	2.40	3.324 (4)	159
C4*B*—H4*B*⋯*Cg*2^i^	0.93	2.88	3.552 (3)	130

Symmetry codes: (i) 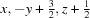; (ii) 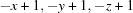; (iii) 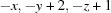.

**Table 2:** Experimental details

Crystal data	
Chemical formula	C_11_H_8_O_5_·C_2_H_6_OS
*M*_r_	298.30
Crystal system, space group	Monoclinic, *P*2_1_/*c*
Temperature (K)	293
*a*, *b*, *c* (Å)	23.1461 (12), 8.2631 (4), 14.6374 (8)
β (°)	97.687 (4)
*V* (Å^3^)	2774.4 (2)
*Z*	8
Radiation type	Mo *K*α
μ (mm^−1^)	0.26
Crystal size (mm)	0.25 × 0.18 × 0.12

Data collection	
Diffractometer	Bruker Kappa APEXII CCD
Absorption correction	Multi-scan (*SADABS*; Bruker, 2008 ▸)
*T*_min_, *T*_max_	0.742, 0.852
No. of measured, independent and observed [*I* > 2σ(*I*)] reflections	25798, 6824, 2743
*R*_int_	0.119
(sin θ/λ)_max_ (Å^−1^)	0.666

Refinement	
*R*[*F* ^2^ > 2σ(*F* ^2^)], *wR*(*F* ^2^), *S*	0.063, 0.208, 0.87
No. of reflections	6824
No. of parameters	376
H-atom treatment	H-atom parameters constrained
Δρ_max_, Δρ_min_ (e Å^−3^)	0.69, −0.42

Computer programs: *APEX2* and *SAINT* (Bruker, 2008 ▸), *SHELXS2018* (Sheldrick, 2008 ▸), *SHELXL2018* (Sheldrick, 2015 ▸), *ORTEP-3 for Windows* and *Mercury* (Macrae *et al.*, 2008 ▸), *WinGX* (Farrugia, 2012 ▸); *Mercury* (Macrae *et al.*, 2008 ▸), *publCIF* (Westrip, 2010 ▸) and *PLATON* (Spek, 2009 ▸).

## supplementary crystallographic information

### Crystal data

**Table:**

C_11_H_8_O_5_·C_2_H_6_OS	*F*(000) = 1248
*M**_r_* = 298.30	*D*_x_ = 1.428 Mg m^−^^3^
Monoclinic, *P*2_1_/*c*	Mo *K*α radiation, λ = 0.71073 Å
*a* = 23.1461 (12) Å	Cell parameters from 6824 reflections
*b* = 8.2631 (4) Å	θ = 1.8–28.3°
*c* = 14.6374 (8) Å	µ = 0.26 mm^−^^1^
β = 97.687 (4)°	*T* = 293 K
*V* = 2774.4 (2) Å^3^	Block, colourless
*Z* = 8	0.25 × 0.18 × 0.12 mm

### Data collection

**Table:**

Bruker Kappa APEXII CCD diffractometer	2743 reflections with *I* > 2σ(*I*)
ω and φ scans	*R*_int_ = 0.119
Absorption correction: multi-scan (*SADABS*; Bruker, 2008)	θ_max_ = 28.3°, θ_min_ = 1.8°
*T*_min_ = 0.742, *T*_max_ = 0.852	*h* = −30→30
25798 measured reflections	*k* = −10→10
6824 independent reflections	*l* = −19→19

### Refinement

**Table:**

Refinement on *F*^2^	0 restraints
Least-squares matrix: full	Hydrogen site location: inferred from neighbouring sites
*R*[*F*^2^ > 2σ(*F*^2^)] = 0.063	H-atom parameters constrained
*wR*(*F*^2^) = 0.208	*w* = 1/[σ^2^(*F*_o_^2^) + (0.1053*P*)^2^] where *P* = (*F*_o_^2^ + 2*F*_c_^2^)/3
*S* = 0.87	(Δ/σ)_max_ < 0.001
6824 reflections	Δρ_max_ = 0.69 e Å^−^^3^
376 parameters	Δρ_min_ = −0.41 e Å^−^^3^

### Special details

**Table:**

Geometry. All esds (except the esd in the dihedral angle between two l.s. planes) are estimated using the full covariance matrix. The cell esds are taken into account individually in the estimation of esds in distances, angles and torsion angles; correlations between esds in cell parameters are only used when they are defined by crystal symmetry. An approximate (isotropic) treatment of cell esds is used for estimating esds involving l.s. planes.

### Fractional atomic coordinates and isotropic or equivalent isotropic displacement parameters (Å^2^)

**Table:**

	*x*	*y*	*z*	*U*_iso_*/*U*_eq_	Occ. (<1)
C13A	−0.04399 (18)	0.4312 (5)	0.3443 (3)	0.0728 (13)	
H13A	−0.070292	0.463777	0.386381	0.109*	
H13B	−0.039226	0.315849	0.346833	0.109*	
H13C	−0.059577	0.462937	0.282872	0.109*	
C13B	0.4520 (2)	0.3039 (6)	0.5340 (4)	0.0918 (16)	
H13D	0.491305	0.265054	0.546953	0.138*	
H13E	0.427430	0.243383	0.569514	0.138*	
H13F	0.450767	0.416386	0.550058	0.138*	
C12B	0.4320 (2)	0.0677 (5)	0.4079 (4)	0.0834 (15)	
H12A	0.419331	0.033276	0.345850	0.125*	
H12B	0.407847	0.018839	0.448683	0.125*	
H12C	0.471795	0.035236	0.425574	0.125*	
S1B	0.42683 (4)	0.27960 (14)	0.41469 (9)	0.0666 (4)	
C8B	0.27020 (14)	0.7563 (4)	0.6754 (2)	0.0410 (9)	
H8B	0.286314	0.852257	0.657309	0.049*	
C8A	0.22217 (14)	1.1613 (4)	0.5296 (2)	0.0388 (8)	
H8A	0.206427	1.062620	0.544340	0.047*	
C1A	0.21016 (13)	1.4456 (4)	0.4892 (2)	0.0343 (8)	
C1B	0.28007 (13)	0.4732 (4)	0.7173 (2)	0.0365 (8)	
C9A	0.18699 (13)	1.2921 (4)	0.5157 (2)	0.0356 (8)	
C6B	0.22279 (14)	0.4729 (4)	0.7336 (2)	0.0374 (8)	
C7B	0.21038 (14)	0.7526 (4)	0.6883 (3)	0.0408 (9)	
C6A	0.26854 (14)	1.4507 (4)	0.4786 (2)	0.0374 (8)	
C9B	0.30397 (13)	0.6232 (4)	0.6888 (2)	0.0350 (8)	
C10B	0.39088 (14)	0.7553 (4)	0.6567 (3)	0.0450 (9)	
H10A	0.365842	0.821170	0.613063	0.054*	
H10B	0.425039	0.726291	0.628436	0.054*	
C7A	0.28261 (15)	1.1714 (4)	0.5224 (3)	0.0425 (9)	
C3A	0.20154 (16)	1.7230 (4)	0.4385 (3)	0.0486 (10)	
H3A	0.179056	1.815309	0.424815	0.058*	
C4A	0.25956 (17)	1.7231 (4)	0.4274 (3)	0.0502 (10)	
H4A	0.275975	1.815759	0.405586	0.060*	
C5A	0.29396 (15)	1.5876 (4)	0.4480 (3)	0.0451 (9)	
H5A	0.333355	1.588921	0.441346	0.054*	
C11B	0.40938 (15)	0.8519 (4)	0.7420 (3)	0.0484 (10)	
C5B	0.19577 (16)	0.3353 (4)	0.7620 (3)	0.0469 (9)	
H5B	0.157087	0.338287	0.772786	0.056*	
C3B	0.28513 (17)	0.1906 (4)	0.7585 (3)	0.0496 (10)	
H3B	0.306236	0.094800	0.767083	0.060*	
C11A	0.08288 (14)	1.0434 (4)	0.4690 (3)	0.0436 (9)	
C4B	0.22748 (17)	0.1949 (4)	0.7737 (3)	0.0501 (10)	
H4B	0.209965	0.101248	0.792202	0.060*	
C2A	0.17680 (15)	1.5856 (4)	0.4699 (2)	0.0423 (9)	
H2A	0.137684	1.586033	0.478387	0.051*	
C2B	0.31131 (15)	0.3286 (4)	0.7305 (2)	0.0429 (9)	
H2B	0.350099	0.325359	0.720247	0.052*	
C12A	0.05788 (19)	0.4705 (5)	0.2783 (3)	0.0771 (14)	
H12D	0.063612	0.355485	0.278054	0.116*	
H12E	0.033342	0.502275	0.223138	0.116*	
H12F	0.094887	0.524054	0.281072	0.116*	
C10A	0.10247 (14)	1.1508 (4)	0.5512 (3)	0.0421 (9)	
H10C	0.068964	1.179268	0.581190	0.050*	
H10D	0.129589	1.091455	0.595162	0.050*	
O2B	0.17604 (10)	0.8632 (3)	0.6759 (2)	0.0583 (7)	
O2A	0.30431 (9)	1.3180 (3)	0.49654 (17)	0.0443 (6)	
O3B	0.36054 (9)	0.6118 (3)	0.67566 (17)	0.0447 (6)	
O1A	0.31786 (11)	1.0636 (3)	0.5349 (2)	0.0623 (8)	
O1B	0.18850 (9)	0.6100 (3)	0.71963 (17)	0.0455 (6)	
O3A	0.13002 (9)	1.2960 (2)	0.52488 (17)	0.0422 (6)	
O4A	0.06268 (12)	0.9068 (3)	0.49773 (19)	0.0586 (8)	
H4A1	0.050295	0.850621	0.453194	0.088*	
O4B	0.43802 (13)	0.9821 (3)	0.7219 (2)	0.0710 (9)	
H4B1	0.445425	1.038184	0.768236	0.106*	
O7B	0.47513 (11)	0.3440 (3)	0.3654 (2)	0.0764 (10)	
O6A	0.01623 (13)	0.6980 (4)	0.3745 (3)	0.0979 (13)	
O5B	0.39977 (12)	0.8143 (4)	0.8178 (2)	0.0682 (9)	
O5A	0.08491 (13)	1.0796 (4)	0.3906 (2)	0.0718 (9)	
S1A	0.02436 (6)	0.52545 (19)	0.37557 (13)	0.0507 (6)	0.782 (5)
S1'A	0.0025 (3)	0.5829 (7)	0.3230 (5)	0.057 (2)	0.218 (5)

### Atomic displacement parameters (Å^2^)

**Table:**

	*U*^11^	*U*^22^	*U*^33^	*U*^12^	*U*^13^	*U*^23^
C13A	0.067 (3)	0.069 (3)	0.085 (4)	−0.020 (2)	0.016 (2)	−0.009 (3)
C13B	0.086 (3)	0.094 (4)	0.090 (4)	−0.016 (3)	−0.010 (3)	−0.007 (3)
C12B	0.088 (3)	0.070 (3)	0.090 (4)	−0.011 (3)	0.001 (3)	0.004 (3)
S1B	0.0449 (6)	0.0689 (7)	0.0854 (10)	0.0084 (5)	0.0070 (6)	0.0063 (6)
C8B	0.0393 (18)	0.0338 (18)	0.051 (3)	−0.0033 (15)	0.0085 (16)	0.0001 (17)
C8A	0.0399 (18)	0.0315 (17)	0.045 (2)	−0.0033 (14)	0.0075 (16)	0.0018 (16)
C1A	0.0360 (17)	0.0355 (18)	0.032 (2)	0.0010 (14)	0.0049 (15)	−0.0044 (15)
C1B	0.0380 (18)	0.0345 (18)	0.036 (2)	−0.0004 (15)	0.0007 (15)	−0.0001 (16)
C9A	0.0353 (17)	0.0366 (19)	0.035 (2)	−0.0010 (14)	0.0033 (15)	−0.0007 (15)
C6B	0.0432 (19)	0.0366 (18)	0.032 (2)	−0.0007 (15)	0.0052 (16)	−0.0041 (16)
C7B	0.0416 (19)	0.039 (2)	0.042 (2)	−0.0008 (16)	0.0051 (16)	−0.0021 (17)
C6A	0.0445 (19)	0.0345 (18)	0.033 (2)	−0.0015 (15)	0.0059 (16)	−0.0057 (16)
C9B	0.0328 (16)	0.0382 (18)	0.033 (2)	0.0004 (14)	0.0028 (15)	−0.0073 (16)
C10B	0.0347 (17)	0.043 (2)	0.059 (3)	−0.0011 (16)	0.0159 (17)	0.0030 (19)
C7A	0.0432 (19)	0.040 (2)	0.044 (2)	0.0032 (16)	0.0033 (17)	−0.0044 (17)
C3A	0.057 (2)	0.041 (2)	0.048 (3)	0.0097 (18)	0.0062 (19)	0.0031 (18)
C4A	0.071 (3)	0.039 (2)	0.041 (3)	−0.0134 (19)	0.011 (2)	0.0038 (18)
C5A	0.047 (2)	0.045 (2)	0.045 (3)	−0.0090 (17)	0.0108 (18)	−0.0043 (18)
C11B	0.0358 (19)	0.042 (2)	0.069 (3)	−0.0054 (16)	0.014 (2)	−0.004 (2)
C5B	0.050 (2)	0.051 (2)	0.040 (3)	−0.0126 (18)	0.0074 (17)	0.0001 (18)
C3B	0.066 (3)	0.038 (2)	0.042 (3)	0.0018 (18)	−0.0043 (19)	0.0061 (18)
C11A	0.0343 (18)	0.044 (2)	0.053 (3)	−0.0022 (16)	0.0095 (18)	0.000 (2)
C4B	0.070 (3)	0.041 (2)	0.038 (3)	−0.0132 (19)	0.0029 (19)	0.0051 (17)
C2A	0.0445 (19)	0.041 (2)	0.041 (2)	0.0018 (16)	0.0038 (17)	0.0004 (17)
C2B	0.0436 (19)	0.042 (2)	0.042 (2)	0.0022 (16)	0.0001 (17)	−0.0009 (17)
C12A	0.070 (3)	0.076 (3)	0.092 (4)	−0.003 (2)	0.034 (3)	−0.030 (3)
C10A	0.0368 (18)	0.0411 (19)	0.050 (3)	−0.0006 (15)	0.0109 (17)	0.0002 (18)
O2B	0.0477 (15)	0.0467 (15)	0.081 (2)	0.0112 (12)	0.0088 (14)	0.0038 (14)
O2A	0.0367 (12)	0.0435 (14)	0.0539 (18)	0.0010 (10)	0.0101 (11)	0.0052 (12)
O3B	0.0342 (13)	0.0378 (13)	0.0629 (19)	−0.0018 (10)	0.0094 (12)	0.0006 (12)
O1A	0.0475 (15)	0.0520 (16)	0.088 (2)	0.0149 (13)	0.0105 (14)	0.0099 (15)
O1B	0.0392 (13)	0.0430 (14)	0.0561 (18)	0.0005 (11)	0.0123 (12)	0.0030 (12)
O3A	0.0340 (12)	0.0348 (13)	0.0583 (18)	0.0003 (10)	0.0085 (11)	0.0034 (11)
O4A	0.0630 (17)	0.0405 (15)	0.072 (2)	−0.0125 (13)	0.0081 (15)	−0.0035 (14)
O4B	0.0748 (19)	0.0550 (17)	0.085 (2)	−0.0232 (15)	0.0173 (18)	−0.0107 (16)
O7B	0.0494 (16)	0.074 (2)	0.110 (3)	0.0176 (14)	0.0238 (16)	0.0302 (18)
O6A	0.074 (2)	0.073 (2)	0.153 (4)	−0.0240 (17)	0.040 (2)	−0.065 (2)
O5B	0.074 (2)	0.074 (2)	0.058 (2)	−0.0207 (15)	0.0138 (16)	−0.0042 (17)
O5A	0.089 (2)	0.079 (2)	0.049 (2)	−0.0262 (17)	0.0148 (17)	−0.0035 (17)
S1A	0.0455 (8)	0.0497 (9)	0.0550 (13)	0.0045 (6)	−0.0008 (7)	−0.0059 (8)
S1'A	0.059 (3)	0.049 (3)	0.061 (5)	0.004 (3)	0.002 (3)	0.007 (3)

### Geometric parameters (Å, º)

**Table:**

C13A—S1'A	1.708 (6)	C10B—H10A	0.9700
C13A—S1A	1.768 (4)	C10B—H10B	0.9700
C13A—H13A	0.9600	C7A—O1A	1.205 (4)
C13A—H13B	0.9600	C7A—O2A	1.384 (4)
C13A—H13C	0.9600	C3A—C2A	1.378 (5)
C13B—S1B	1.777 (5)	C3A—C4A	1.374 (5)
C13B—H13D	0.9600	C3A—H3A	0.9300
C13B—H13E	0.9600	C4A—C5A	1.384 (5)
C13B—H13F	0.9600	C4A—H4A	0.9300
C12B—S1B	1.759 (5)	C5A—H5A	0.9300
C12B—H12A	0.9600	C11B—O5B	1.201 (5)
C12B—H12B	0.9600	C11B—O4B	1.317 (4)
C12B—H12C	0.9600	C5B—C4B	1.372 (5)
S1B—O7B	1.506 (3)	C5B—H5B	0.9300
C8B—C9B	1.348 (4)	C3B—C2B	1.379 (5)
C8B—C7B	1.423 (4)	C3B—C4B	1.383 (5)
C8B—H8B	0.9300	C3B—H3B	0.9300
C8A—C9A	1.352 (4)	C11A—O5A	1.193 (4)
C8A—C7A	1.420 (4)	C11A—O4A	1.312 (4)
C8A—H8A	0.9300	C11A—C10A	1.515 (5)
C1A—C6A	1.382 (4)	C4B—H4B	0.9300
C1A—C2A	1.399 (4)	C2A—H2A	0.9300
C1A—C9A	1.449 (4)	C2B—H2B	0.9300
C1B—C6B	1.378 (4)	C12A—S1'A	1.776 (7)
C1B—C2B	1.396 (4)	C12A—S1A	1.769 (4)
C1B—C9B	1.441 (5)	C12A—H12D	0.9600
C9A—O3A	1.344 (4)	C12A—H12E	0.9600
C6B—C5B	1.388 (5)	C12A—H12F	0.9600
C6B—O1B	1.383 (4)	C10A—O3A	1.436 (4)
C7B—O2B	1.209 (4)	C10A—H10C	0.9700
C7B—O1B	1.384 (4)	C10A—H10D	0.9700
C6A—C5A	1.377 (5)	O4A—H4A1	0.8200
C6A—O2A	1.378 (4)	O4B—H4B1	0.8200
C9B—O3B	1.352 (3)	O6A—S1'A	1.229 (6)
C10B—O3B	1.424 (4)	O6A—S1A	1.438 (3)
C10B—C11B	1.496 (5)		
			
S1A—C13A—H13A	109.5	O2A—C7A—C8A	118.0 (3)
S1A—C13A—H13B	109.5	C2A—C3A—C4A	119.8 (3)
H13A—C13A—H13B	109.5	C2A—C3A—H3A	120.1
S1A—C13A—H13C	109.5	C4A—C3A—H3A	120.1
H13A—C13A—H13C	109.5	C3A—C4A—C5A	121.1 (3)
H13B—C13A—H13C	109.5	C3A—C4A—H4A	119.4
S1B—C13B—H13D	109.5	C5A—C4A—H4A	119.4
S1B—C13B—H13E	109.5	C6A—C5A—C4A	118.5 (3)
H13D—C13B—H13E	109.5	C6A—C5A—H5A	120.8
S1B—C13B—H13F	109.5	C4A—C5A—H5A	120.8
H13D—C13B—H13F	109.5	O5B—C11B—O4B	125.3 (4)
H13E—C13B—H13F	109.5	O5B—C11B—C10B	124.4 (3)
S1B—C12B—H12A	109.5	O4B—C11B—C10B	110.3 (4)
S1B—C12B—H12B	109.5	C4B—C5B—C6B	118.4 (3)
H12A—C12B—H12B	109.5	C4B—C5B—H5B	120.8
S1B—C12B—H12C	109.5	C6B—C5B—H5B	120.8
H12A—C12B—H12C	109.5	C2B—C3B—C4B	119.9 (3)
H12B—C12B—H12C	109.5	C2B—C3B—H3B	120.0
O7B—S1B—C12B	105.3 (2)	C4B—C3B—H3B	120.0
O7B—S1B—C13B	105.3 (2)	O5A—C11A—O4A	125.8 (4)
C12B—S1B—C13B	98.7 (2)	O5A—C11A—C10A	124.8 (3)
C9B—C8B—C7B	121.3 (3)	O4A—C11A—C10A	109.3 (4)
C9B—C8B—H8B	119.4	C5B—C4B—C3B	120.9 (3)
C7B—C8B—H8B	119.4	C5B—C4B—H4B	119.6
C9A—C8A—C7A	121.4 (3)	C3B—C4B—H4B	119.6
C9A—C8A—H8A	119.3	C3A—C2A—C1A	120.4 (3)
C7A—C8A—H8A	119.3	C3A—C2A—H2A	119.8
C6A—C1A—C2A	118.3 (3)	C1A—C2A—H2A	119.8
C6A—C1A—C9A	117.4 (3)	C3B—C2B—C1B	120.6 (3)
C2A—C1A—C9A	124.3 (3)	C3B—C2B—H2B	119.7
C6B—C1B—C2B	117.8 (3)	C1B—C2B—H2B	119.7
C6B—C1B—C9B	117.8 (3)	S1A—C12A—H12D	109.5
C2B—C1B—C9B	124.3 (3)	S1A—C12A—H12E	109.5
O3A—C9A—C8A	125.7 (3)	H12D—C12A—H12E	109.5
O3A—C9A—C1A	114.1 (3)	S1A—C12A—H12F	109.5
C8A—C9A—C1A	120.2 (3)	H12D—C12A—H12F	109.5
C1B—C6B—C5B	122.4 (3)	H12E—C12A—H12F	109.5
C1B—C6B—O1B	121.3 (3)	O3A—C10A—C11A	111.9 (3)
C5B—C6B—O1B	116.3 (3)	O3A—C10A—H10C	109.2
O2B—C7B—O1B	115.5 (3)	C11A—C10A—H10C	109.2
O2B—C7B—C8B	126.6 (3)	O3A—C10A—H10D	109.2
O1B—C7B—C8B	117.9 (3)	C11A—C10A—H10D	109.2
C5A—C6A—O2A	116.3 (3)	H10C—C10A—H10D	107.9
C5A—C6A—C1A	121.9 (3)	C6A—O2A—C7A	121.2 (3)
O2A—C6A—C1A	121.7 (3)	C9B—O3B—C10B	118.8 (2)
C8B—C9B—O3B	126.1 (3)	C7B—O1B—C6B	121.2 (3)
C8B—C9B—C1B	120.4 (3)	C9A—O3A—C10A	118.9 (2)
O3B—C9B—C1B	113.4 (3)	C11A—O4A—H4A1	109.5
O3B—C10B—C11B	112.0 (3)	C11B—O4B—H4B1	109.5
O3B—C10B—H10A	109.2	O6A—S1A—C12A	108.5 (2)
C11B—C10B—H10A	109.2	O6A—S1A—C13A	108.8 (2)
O3B—C10B—H10B	109.2	C12A—S1A—C13A	98.8 (2)
C11B—C10B—H10B	109.2	O6A—S1'A—C13A	124.9 (5)
H10A—C10B—H10B	107.9	O6A—S1'A—C12A	119.4 (5)
O1A—C7A—O2A	115.2 (3)	C13A—S1'A—C12A	100.8 (3)
O1A—C7A—C8A	126.8 (3)		
			
C7A—C8A—C9A—O3A	−177.0 (3)	O3B—C10B—C11B—O5B	−0.3 (5)
C7A—C8A—C9A—C1A	2.4 (5)	O3B—C10B—C11B—O4B	179.0 (3)
C6A—C1A—C9A—O3A	179.9 (3)	C1B—C6B—C5B—C4B	−0.2 (5)
C2A—C1A—C9A—O3A	−3.1 (5)	O1B—C6B—C5B—C4B	177.8 (3)
C6A—C1A—C9A—C8A	0.5 (5)	C6B—C5B—C4B—C3B	0.6 (6)
C2A—C1A—C9A—C8A	177.5 (3)	C2B—C3B—C4B—C5B	−0.4 (6)
C2B—C1B—C6B—C5B	−0.2 (5)	C4A—C3A—C2A—C1A	−1.0 (6)
C9B—C1B—C6B—C5B	−179.4 (3)	C6A—C1A—C2A—C3A	1.9 (5)
C2B—C1B—C6B—O1B	−178.2 (3)	C9A—C1A—C2A—C3A	−175.0 (3)
C9B—C1B—C6B—O1B	2.6 (5)	C4B—C3B—C2B—C1B	0.0 (5)
C9B—C8B—C7B—O2B	−178.0 (4)	C6B—C1B—C2B—C3B	0.3 (5)
C9B—C8B—C7B—O1B	3.1 (5)	C9B—C1B—C2B—C3B	179.5 (3)
C2A—C1A—C6A—C5A	−1.4 (5)	O5A—C11A—C10A—O3A	−6.4 (5)
C9A—C1A—C6A—C5A	175.8 (3)	O4A—C11A—C10A—O3A	174.6 (3)
C2A—C1A—C6A—O2A	179.8 (3)	C5A—C6A—O2A—C7A	−176.2 (3)
C9A—C1A—C6A—O2A	−3.1 (5)	C1A—C6A—O2A—C7A	2.8 (5)
C7B—C8B—C9B—O3B	177.7 (3)	O1A—C7A—O2A—C6A	179.0 (3)
C7B—C8B—C9B—C1B	−0.3 (5)	C8A—C7A—O2A—C6A	0.2 (5)
C6B—C1B—C9B—C8B	−2.6 (5)	C8B—C9B—O3B—C10B	8.3 (5)
C2B—C1B—C9B—C8B	178.2 (3)	C1B—C9B—O3B—C10B	−173.6 (3)
C6B—C1B—C9B—O3B	179.2 (3)	C11B—C10B—O3B—C9B	78.5 (4)
C2B—C1B—C9B—O3B	0.0 (5)	O2B—C7B—O1B—C6B	177.8 (3)
C9A—C8A—C7A—O1A	178.7 (4)	C8B—C7B—O1B—C6B	−3.1 (5)
C9A—C8A—C7A—O2A	−2.7 (5)	C1B—C6B—O1B—C7B	0.3 (5)
C2A—C3A—C4A—C5A	−0.6 (6)	C5B—C6B—O1B—C7B	−177.8 (3)
O2A—C6A—C5A—C4A	178.8 (3)	C8A—C9A—O3A—C10A	−0.4 (5)
C1A—C6A—C5A—C4A	−0.1 (5)	C1A—C9A—O3A—C10A	−179.8 (3)
C3A—C4A—C5A—C6A	1.1 (6)	C11A—C10A—O3A—C9A	−84.3 (3)

### Hydrogen-bond geometry (Å, º)

*Cg*1 is the centroid of the C1*A*–C6*A* ring.

**Table:**

*D*—H···*A*	*D*—H	H···*A*	*D*···*A*	*D*—H···*A*
O4*A*—H4*A*1···O6*A*	0.82	1.82	2.621 (5)	167
O4*B*—H4*B*1···S1*B*^i^	0.82	2.70	3.479 (3)	159
O4*B*—H4*B*1···O7*B*^i^	0.82	1.78	2.595 (4)	169
C10*B*—H10*A*···O1*A*	0.97	2.49	3.423 (4)	161
C10*B*—H10*B*···O7*B*^ii^	0.97	2.37	3.266 (4)	153
C10*A*—H10*C*···O6*A*^iii^	0.97	2.38	3.330 (5)	165
C10*A*—H10*D*···O2*B*	0.97	2.40	3.324 (4)	159
C4*B*—H4*B*···*Cg*1^i^	0.93	2.88	3.552 (3)	130

Symmetry codes: (i) *x*, −*y*+3/2, *z*+1/2; (ii) −*x*+1, −*y*+1, −*z*+1; (iii) −*x*, −*y*+2, −*z*+1.
